# Oral tranexamic acid for acute management of active bleeding from iris microhemangiomatosis: A case report

**DOI:** 10.1016/j.ajoc.2024.102000

**Published:** 2024-01-19

**Authors:** Dario Marangoni, Antonio Gemito, Serena Milan, Daniele Tognetto

**Affiliations:** Eye Clinic, Department of Medical Surgical Sciences and Health, University of Trieste, 34129, Trieste, Italy

**Keywords:** Iris microhemangiomatosis, Tranexamic acid, Intraocular bleeding

## Abstract

**Purpose:**

to report a case of active intraocular bleeding caused by iris microhemangiomatosis managed with oral tranexamic acid.

**Observations:**

an 80-year-old male was referred to our emergency department for acute intraocular bleeding. Eye exam showed filiform bleeding arising from a cluster of vascular tufts at the upper pupillary margin, which was consistent with a diagnosis of iris microhemangiomatosis. The bleeding had started 6 hours before and could not be halted by conservative maneuvers such as ocular compression and application of sympathomimetic drops. Oral tranexamic acid 500 mg was administered and led to prompt resolution of the hemorrhage within 60 minutes. The patient was monitored for 3 months and showed no recurrence of the hemorrhage.

**Conclusion and importance:**

oral tranexamic acid may represent a viable option to manage active intraocular bleeding from iris microhemangiomatosis, facilitating rapid hemorrhage resolution.

## 1 Background

Iris microhemangiomas, also referred to as Cobb's tufts, are unilateral or bilateral hamartomas of the iris stromal blood vessels, first described by Fechner in 1958.^1^ While these lesions are often asymptomatic and found incidentally on a routine eye exam, they can sometimes cause significant visual disturbances due to persistent intraocular bleeding and subsequent increase in intraocular pressure (IOP).^2^ Since most hemorrhages resolve spontaneously and result in low grade hyphema, patients are usually managed conservatively with anti-inflammatory and cycloplegic drops, which are combined with hypotensive agents if IOP is elevated. However, because the duration of the intraocular bleeding is unpredictable, a close observation of the patient is warranted until the bleed completely resolves. Here, we report a case of an intraocular bleeding caused by iris microhemangiomatosis which resolved after the administration of oral tranexamic acid, leading to prompt discharge of the patient.

## 2 Case report

An 80-year-old male was referred to our ophthalmic emergency department from a local ophthalmologist due to active intraocular bleeding in his right eye. On presentation, the patient complained of progressively worsening blurry vision in his right eye that had started 6 hours before. He denied any ocular trauma or recent use of eye drops. Past ophthalmic history was uneventful. His past medical history was significant for systemic hypertension on lisinopril and diet-controlled diabetes mellitus. Ten years before, he had been diagnosed with non-metastatic colon cancer, for which he underwent right hemicolectomy and adjuvant chemotherapy with oxaliplatin and capecitabine, resulting in complete remission.

**Fig. 1:**
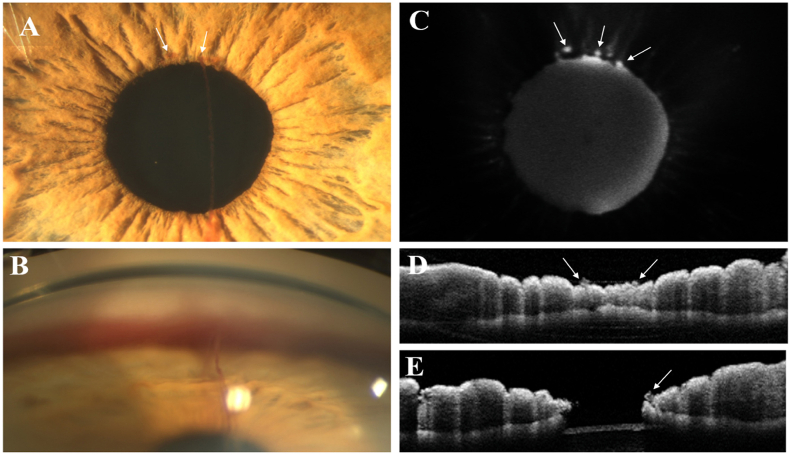
Multimodal imaging of iris microhemangiomas. A) Anterior segment photography of the right eye showing two vascular tufts (arrows), with a linear vertical blood streak coming from the right lesion due to active bleeding. B) Gonioscopy of the inferior sector demonstrating the filiform bleeding and blood deposition. C) Fluorescein iridography (early phases) reveals two hyperfluorescent spots corresponding to the vascular tufts observed on biomicroscopy and an additional hyperfluorescent lesion at 1 o'clock. D), E) Anterior segment OCT showing 3 distinct nodular growths on the iris epithelium surface corresponding to the vascular tufts (arrows).

On eye exam, best-corrected visual acuity was 20/25 in the right eye and 20/20 in the left eye. IOP was 18 mmHg in both eyes. Pupillary reflexes were normal. Slit lamp exam of the right eye showed an active filiform bleeding which originated from the upper pupillary margin and +2 red blood cells in the anterior chamber. At higher magnification, two coils of clustered minute blood vessels were visualized at the iris pupillary margin at 11 and 12 o'clock (Fig. 1A). Iris morphology was otherwise normal without any pupillary margin distortion or neovascularization. The anterior lens capsule was covered by a linear vertical blood streak and lens showed mild nuclear opacities. Gonioscopy showed deposition of blood in the inferior sector (Fig. 1B), preventing visualization of the inferior angle structures. The angle was otherwise wide-open in the other sectors, without any signs of neovascularization. Slit lamp exam of the left eye was unremarkable except for a mild age-related lens nuclear opacity. Fundus examination was normal in both eyes. There were no signs of retinal vascular diseases, including retinal microaneurysms, hemorrhages, vessel tortuosity and dilation, or any signs of disc or retinal neovascularization. No signs of intraocular inflammation, including vitreous cells or haze, were observed. Based on these clinical findings, the diagnosis of iris microhemangiomas was suspected.

Due to the persistence of intraocular bleeding, we initially tried to constrict the vessels and halt the bleed by applying a continuous pressure to the eye with a 3-mirror lens for 10 minutes and by administering mydriatic sympathomimetic drops (Phenylephrine 10 %). However, despite the treatment, the hemorrhage increased in intensity during the following hour and we endeavored to halt the bleeding by administering tranexamic acid 500 mg oral solution, a blood clot stabilizer. Sixty minutes after a single dose of tranexamic acid, the hemorrhage completely stopped and after 3 hours of observation the patient was discharged home on topical steroids and cycloplegic agents with the recommendation to keep a semi-reclined position. No adverse effects were observed after the administration of tranexamic acid.

A fluorescein iridography was performed the following day and showed three hyperfluorescent spots in proximity to the upper pupillary ruff of the right eye between 11 and 1 o'clock (Fig. 1C), which remained stable in size until the late angiogram phases. There were no remarkable angiographic findings in the left eye. To rule out any sign of retinal ischemia or neovascularization, a fluorescein angiography was ordered and was normal in both eyes. Anterior segment OCT of the iris revealed three distinct nodular growths on the iris epithelium surface, corresponding to the hyperfluorescent spots observed on iris angiography (Fig. 1. D, E). The patient was monitored for the following 3 months and showed no recurrence of intraocular bleeding or any change in visual acuity and IOP.

## 3 Discussion

Tranexamic acid has been proposed for the management of different eye conditions, including traumatic hyphema,^3^ diabetic retinopathy and retinal vein occlusion,^4^ and prevention of vitreous hemorrhage after vitreoretinal surgery.^5^ Here, we describe a case of intraocular bleed caused by iris microhemangiomatosis, which was managed with the use of oral tranexamic acid.

Iris microhemangiomas are acquired benign vascular lesions that consist in multiple small vascular dilations, typically near the pupillary margin. Most cases remain asymptomatic, but rarely, they can present with spontaneous or trauma-induced intraocular bleeding and be complicated by various degrees of vision loss and IOP increase, depending on the severity of the hemorrhage.^6^ While many cases resolve within 48 hours even without treatment,^7^ there are reports of persistent hemorrhage associated with total hyphema and IOP higher than 50 mmHg which can be sight-threatening.^8^ Therefore, it is crucial to halt the bleeding in a timely manner to avoid such complications.

The application of ocular compression by gonioscopy or pressure patch^9^ and mydriatic eyedrops have been proposed to promote hemostasis in the early phases of the bleeding.^7^ However, this approach was not successful in our case. Alternatively, laser photocoagulation of the telangiectatic vessels may be used to stop the active bleeding^7^^,^^10^, ^11^ but the usual localization of the vascular lesions at the pupillary ruff increases the chances to damage the lens during the procedure, among other laser-associated risks. Moreover, the availability of argon laser may be limited.

In our patient, the administration of tranexamic acid completely halted the hemorrhage after about 1 hour. Because bleedings associated with iris microhemangiomatosis can resolve spontaneously, we cannot rule out that the hemorrhage would have stopped even without the use of tranexamic acid. However, based on its pharmaceutical properties, we hypothesize that the medication contributed, at least, to its resolution.

Tranexamic acid is a molecular analogue of lysine which reduces bleeding by inhibiting fibrinolysis and preventing blood clots plasmin-mediated lysis.^12^^,^^13^ Previous studies have demonstrated that oral administration of tranexamic acid at standard doses, ranging from 2 to 3.9 g daily,^14^^,^^15^ can halt or decrease bleeding from peripheral vessels in patients with menorrhagia or epistaxis. These dosages are supported by pharmacokinetics studies *in vivo*, which have shown that, when given orally at the dose of 2000 mg or 25 mg/Kg, tranexamic acid produces near-maximal inhibition of fibrinolysis, by reaching a serum concentration of 10 mg/L after about 60 minutes.^12^^,^^16^ Of note, in our patient the bleeding completely halted about 1 hour after oral administration of 500 mg of tranexamic acid, a dose which was 4 times smaller than the dose that produces a significant inhibition of fibrinolysis in 1 hour.

We speculate that while a serum concentration of 10 mg/L or greater is necessary to reduce large blood loss, such as post-traumatic hemorrhages,^17^ lower levels of fibrinolysis inhibition, and therefore a lower serum concentration of tranexamic acid, may have been sufficient to accelerate the resolution of a minor bleed like in our patient. Iris microhemangiomatosis are lesions of about 150 μm in size and contain vessels of 15–25 μm diameter.^1^^,^^18^ Given their small size, the blood flow deriving from their rupture is minimal and not remotely comparable to the amount of bleeding that originates from other peripheral vessels or vascular plexi, for which standard doses of tranexamic acid are used in the clinical practice.^14^^,^^15^ In addition, Picetti et al.,^13^ showed that partial inhibition of fibrinolysis can be achieved also with lower plasmatic concentrations of tranexamic acid i.e., between 5 and 10 mg/L. Furthermore, the therapeutic concentration that was found to produce at least 80 % of fibrinolysis inhibition, i.e., 10 mg/dL, derives from *in-vitro* studies, and it has been suggested that it may overestimate the effective serum concentration that is required to inhibit fibrinolysis *in-vivo*.^13^

A study by Bramsen^16^ shows that the aqueous humor concentration of tranexamic acid after a single oral dose of 25 mg/kg, which is about 4 times larger than the dose that we dispensed to our patient, is 0.73 mg/L after 1 hour, indicating that tranexamic acid can penetrate the blood-aqueous barrier of the irideal vessel and access a privileged space such as the anterior chamber. Although, to our knowledge, there are no data on the minimum concentration of tranexamic acid necessary to inhibit fibrinolysis in the aqueous humor. Tovi et al.^19^ found that a concentration of 0.3 mg/L in the cerebrospinal fluid significantly reduced in 30 minutes the content of fibrin degradation products, which are indirect indicators of fibrinolysis. In those cases where the blood-aqueous barrier is defective, e.g., after ocular trauma or in the presence of ruptured telangiectatic vessels like in our patients, it can be assumed that a larger concentration of tranexamic acid may pass into the anterior chamber and, therefore, that also lower oral doses may be enough to stabilize clots in the event of minimal bleedings.

In terms of safety, no adverse events were observed in our patient during the following 3 months. Tranexamic acid has a favorable safety profile and is generally well-tolerated by most patients at standard doses, but due to some concerns for potentials thrombotic events and lower urinary excretion in case of renal impairment, it should be used cautiously in patients with a history of thromboembolic disease or renal dysfunction, and careful patient selection is thus required.^20^

## 4 Conclusions

Tranexamic acid may represent a possible therapeutic option to facilitate the arrest of intraocular bleeding caused by iris microhemangiomatosis and reduce the incidence of further complications. Prompt hemostasis achievement could permit patient discharge in a safe and cost-effective manner. Further studies are warranted to determine the optimal dosage and route of administration of tranexamic acid to efficiently address this condition.

### 4.1 Patient consent

Written consent to publish personal information and case details has been obtained from the patient.

## CRediT authorship contribution statement

**Dario Marangoni:** Writing – review & editing, Writing – original draft, Conceptualization. **Antonio Gemito:** Writing – review & editing, Conceptualization. **Serena Milan:** Writing – review & editing. **Daniele Tognetto:** Writing – review & editing, Supervision.

## Declaration of competing interest

The authors declare that they have no known competing financial interests or personal relationships that could have appeared to influence the work reported in this paper.
